# Feasibility of Early Vestibular Screening and Developmental Changes in Air- and Bone-Conducted Cervical Vestibular Evoked Myogenic Potentials in Infants and Children with Normal Hearing

**DOI:** 10.3390/audiolres15030067

**Published:** 2025-06-09

**Authors:** Jiali Shen, Xiaobao Ma, Lu Wang, Wei Wang, Jianyong Chen, Qing Zhang, Maoli Duan, Yulian Jin, Jun Yang

**Affiliations:** 1 Department of Otorhinolaryngology-Head and Neck Surgery, Xinhua Hospital, Shanghai Jiaotong University School of Medicine, Shanghai 200025, China; shenjiali@xinhuamed.com.cn (J.S.);; 2 Shanghai Jiaotong University School of Medicine Ear Institute, Shanghai 200025, China; 3 Shanghai Key Laboratory of Translational Medicine on Ear and Nose Diseases, Shanghai 200011, China; 4 Ear Nose and Throat Patient Area, Trauma and Reparative Medicine Theme, Karolinska University Hospital, 17176 Stockholm, Sweden; 5 Division of Ear, Nose, and Throat Diseases, Department of Clinical Science, Intervention and Technology, Karolinska Institutet, 17177 Stockholm, Sweden

**Keywords:** cervical vestibular evoked myogenic potential, normal hearing, infants and children, age-related changes, vestibular screening

## Abstract

**Objective:** To evaluate the feasibility of vestibular screening in infants and investigate age-related changes in the characteristics of air-conducted sound cervical vestibular evoked myogenic potential (ACS-cVEMP) and bone-conducted vibration cervical vestibular evoked myogenic potential (BCV-cVEMP) in infants and children with normal hearing, aiming to provide new insights into the developmental trajectory of vestibular function during early childhood. **Methods:** A total of 159 subjects aged 3 months to 17 years old were divided into seven age groups. Additionally, 20 adults aged 18–30 years were included as controls to explore developmental changes in the sacculocollic reflex pathway. **Results:** The response rates of BCV-cVEMP in 3-month-olds were significantly higher than that of ACS-cVEMP (*p* = 0.048), while no significant difference was observed in other age groups (*p* > 0.05). Age-related changes were found in both latencies and amplitudes of ACS-cVEMP and BCV-cVEMP. ACS-cVEMP latencies reached adult levels at 13–17 years, while BCV-cVEMP latencies normalized by 7–12 years. ACS-cVEMP amplitudes increased with age, stabilizing at 4 years, whereas BCV-cVEMP amplitudes peaked at 4–6 years before gradually decreasing. **Conclusions:** This study demonstrates that cVEMP is not only a viable tool for vestibular screening in infants but also reveals crucial age-related developmental changes in the vestibular system. These findings contribute new insights into the maturation of the vestibular reflex pathways and provide normative data that can be used to guide early vestibular screening practices.

## 1. Introduction

The vestibular system, which includes the semicircular canals and otolithic organs, is one of the earliest sensory systems to develop and plays a vital role in balance and spatial orientation [1,2]. The neonatal and infant stages are critical for developing motor skills, visual stability, and postural control, which are closely tied to vestibular function [3,4].

Assessing vestibular function in infants is challenging due to their limited ability to cooperate. Common assessment methods include the Video Head Impulse Test (vHIT), Caloric Test (CT), Rotary Chair Test (RCT), Cervical Vestibular Evoked Myogenic Potential (cVEMP), Ocular Vestibular Evoked Myogenic Potential (oVEMP), Subjective Visual Vertical (SVV), and Subjective Visual Horizontal (SVH) tests, etc. However, many of these require high levels of cooperation, limiting their applicability for infants [5].

Among these, cVEMP is particularly suitable for infants. It is a non-invasive, objective measure of the saccular and inferior vestibular nerve function. Importantly, cVEMP can be reliably recorded in infants as young as 3 months of age, whereas oVEMP are typically feasible only in children older than 2 years, due to their more complex recording requirements and lower response rates in younger populations [6,7]. Studies have demonstrated the feasibility of using cVEMP in infants, showing that waveform characteristics are generally similar to those of adults, though with notable differences such as shorter latency and greater amplitude variability [8,9]. Additionally, cVEMP is advantageous due to its availability in Auditory Brainstem Response (ABR) testing equipment and its established correlation with motor abilities, making it valuable for early detection of vestibular dysfunction and motor delays [3,4,10,11,12,13,14]. Importantly, cVEMP testing plays a critical role in evaluating vestibular function in children at risk for balance disorders, such as those with congenital cytomegalovirus (CMV) infection and has been recommended as part of early screening protocols for such populations [15].Despite these advantages, vestibular evaluation in pediatric populations remains largely limited to older children with severe and profound hearing loss, cochlear implant candidates, or children with diagnosed balance disorders, and is rarely applied in early infancy [12,13,14]. In addition, cVEMP parameters, such as latencies, amplitudes, and interaural asymmetry ratios (IAR), vary with age, yet there is a lack of normative data for infants and young children [2,4,8,16,17,18,19,20]. Previous studies often categorized infants and children into broad age groups, limiting the precision needed for clinical assessment [21,22].

The present study aims to explore age-related alterations and establish reference values for ACS-cVEMP and BCV-cVEMP latencies, corrected amplitudes, and IARs in infants and children with normal hearing. These data are essential for understanding vestibular development in early life and form a foundation for diagnosing and intervening in vestibular dysfunction.

## 2. Materials and Methods

### 2.1. Subjects

A retrospective analysis was conducted on 159 subjects aged 3 months to 17 years old with bilateral normal hearing who visited our department between May 2021 and December 2023. All subjects were divided into seven age groups: 3 months, 4–6 months, 7–12 months, 1–3 years old, 4–6 years old, 7–12 years old, and 13–17 years old. In addition, 20 subjects aged 18–30 years old with normal hearing were collected as controls. The distribution of age, gender, and number of subjects in each group is shown in Table 1.

### 2.2. Methods

#### 2.2.1. ACS-cVEMP, BCV-cVEMP Measurement

All infants’ and children’s parents and healthy adults signed the informed consent. All subjects accepted ACS-cVEMP and BCV-cVEMP while awake. The measurements were conducted in a randomized sequential order using the Eclipse device (Interacoustics, Denmark). Detailed information on the specific test equipment and methods refers to our previous article [4]. During the test, infants should be fully awake and in a supine position. The skin of the electrode site is prepared with 75% alcohol and scrubbed gently before placing the electrode. Active electrodes are positioned at the upper third of the bilateral SCMM, a reference electrode on the suprasternal notch, and a ground electrode on the forehead for both tests. The electrode impedance must be less than 5 kΩ and the impedance between the electrodes is approximately equal [3,4,13,23]. One audiologist manages the software, while another gently rotates the infant’s head to the opposite side to ensure the SCMM is fully contracted, aiming to have the chin touch the shoulder as closely as possible. A family member or caregiver assists by comforting the infant and applying gentle pressure to the infant’s shoulder to prevent lifting. Toys and videos can be used to distract the infant. A minimum of two trials per side is necessary to ensure waveform repeatability and the accuracy of the test results.

This study was approved by the Ethics Committee of Xinhua Hospital Affiliated to Shanghai Jiaotong University School of Medicine (Approval No. XHEC-D-2022-138. Code: XHEC-D-2022-138). All procedures were conducted in strict accordance with the ethical principles outlined in the Declaration of Helsinki (2013 revision), and written informed consent was obtained from all participants’ legal guardians prior to their involvement in the research.

#### 2.2.2. Audiological Assessment

All subjects completed otoscopy, tympanometry, distortion product otoacoustic emissions (DPOAE), click-evoked Auditory Brainstem Response (click-ABR) and Tone-Burst ABR (TB-ABR) at 500 Hz and 1000 Hz under sedation. Specific test methods and explanation of results refer to our previous article [4].

#### 2.2.3. Inclusion and Exclusion Criteria

All infants included in this study were born at full term (≥37 weeks of gestation) without complications. Criteria for normal hearing in infants and children below 3 years old: (1) no abnormalities in otoscopy, type A tympanogram at 226 Hz and 1000 Hz probe tone in both ears; (2) passed DPOAE (all frequency points from 500 Hz to 8 kHz passed with signal-to-noise ratio ≥ 6 dB and signal strength within the normal range); (3) click-ABR response threshold within 30 dB nHL; (4) no developmental abnormalities observed during routine follow-up assessments in the pediatric care department after birth.

Inclusion criteria for subjects with normal hearing over 3 years old: (1) No history of ear or vestibular system disease, no symptoms of tinnitus or dizziness; (2) Normal otoscopy and tympanometry, pure-tone audiometry thresholds within 20 dB HL at 250–8000 Hz.

Exclusion Criteria: Children with congenital CMV infection, recent head trauma, exposure to loud noises, ototoxic drug use, or a history of otitis media with effusion (OME) were excluded. A neurological screening and nystagmus assessment were performed in the department of child healthcare for all participants to exclude those with potential central or vestibular pathologies.

### 2.3. Statistical Analysis

Data were processed using SPSS 26.0 statistical software (Chicago, IL, USA). Continuous data were described using mean ± standard deviation or interquartile range. Chi-square tests and Fisher’s exact tests were used to compare response rates between groups. Welch’s analysis of variance was used to compare ACS-cVEMP and BCV-cVEMP latencies and interpeak intervals between groups, and the Wilcoxon rank-sum test was used to compare corrected amplitudes between groups. The non-parametric Mann–Whitney test was used to compare the change in amplitude before and after correction. The significance level was set at α = 0.05, with *p* < 0.05 indicating statistical significance.

## 3. Results

### 3.1. The Response Rates and Waveforms of ACS-cVEMP and BCV-cVEMP in Different Age Groups with Normal Hearing

**Figure 1:**
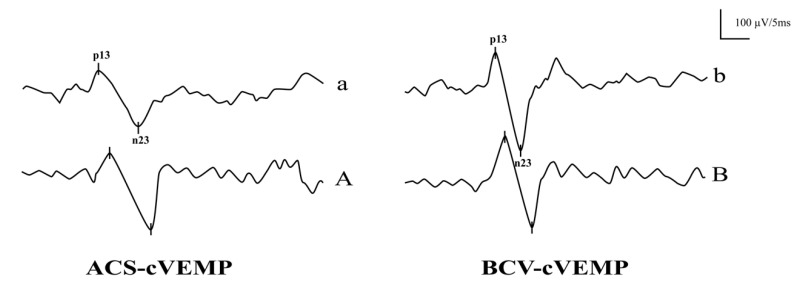
Representative Raw ACS-cVEMP and BCV-cVEMP waveforms of a 3-month-old infant and a 20-year-old adult with bilateral normal hearing. a ACS-cVEMP waveform of a 3-month-old infant with normal hearing. b BCV-cVEMP waveform of a 3-month-old infant with normal hearing. A ACS-cVEMP waveform of a 20-year-old adult with normal hearing. B BCV-cVEMP waveform of a 20-year-old adult with normal hearing.

The response rates of ACS-cVEMP varied significantly across age groups with normal hearing (*p* < 0.001), though Bonferroni correction showed no statistically significant difference among individual groups (*p* > 0.05). No significant difference was observed in BCV-cVEMP response rates across age groups (*p* = 0.417). Within the same age group, BCV-cVEMP response rates were significantly higher than ACS-cVEMP in the 3-month-old group (*p* = 0.048), while no significant difference was found in the 4–6 month and 7–12 month groups (*p* = 0.269 and *p* = 0.494, respectively). For subjects over one year, both stimuli yielded 100% response rates. Figure 1 shows raw ACS-cVEMP and BCV-cVEMP waveforms in a 3-month-old infant (panels a and b) and a 20-year-old adult (panels A and B), both with normal hearing.

### 3.2. ACS-cVEMP Latency in Different Age Groups with Normal Hearing

**Figure 2:**
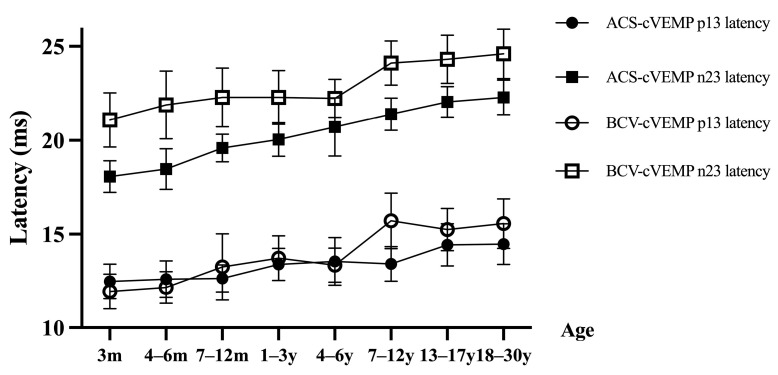
Latencies of ACS-cVEMP and BCV-cVEMP among different age groups with normal hearing. Solid black circles represent p13 latency of ACS-cVEMP. Solid black squares represent n23 latency of ACS-cVEMP. Open circles represent p13 latency of BCV-cVEMP. Open squares represent n23 latency of BCV-cVEMP. The p13 and n23 latencies of ACS-cVEMP and BCV-cVEMP gradually increase with age in different age groups with normal hearing.

Significant differences were observed in ACS-cVEMP p13 and n23 latencies across age groups (both *p* < 0.001), with latencies increasing with age (Figure 2). Bonferroni-corrected comparisons indicated no significant p13 latency difference among the 3, 4–6, and 7–12 month groups (all *p* > 0.05); however, these latencies were significantly shorter than those in groups aged over 1 year (all *p* < 0.05). For the 1–3, 4–6, and 7–12 year groups, latencies remained significantly shorter than those in groups aged 13+ years (all *p* < 0.05). No significant difference was found in latencies for ages 13–17 compared to adults. For individuals under 13, p13 latency was consistently shorter than adult values. Similarly, n23 latency showed no significant difference between the 3 and 4–6 month groups (*p* > 0.05), though latencies were significantly shorter than those in groups aged 7 months and older (all *p* < 0.05). No significant difference was noted between the 7–12 months and 1–3 year groups, nor between the 1–3 and 4–6 year groups (all *p* > 0.05), but latencies were consistently shorter than those of groups aged 7+ years (all *p* < 0.05). Under age 13, n23 latency remained shorter than in adults, gradually increasing with age (Table 2).

### 3.3. BCV-cVEMP Latency in Different Age Groups with Normal Hearing

The p13 and n23 latencies of BCV-cVEMP also showed significant age-related differences (both *p* < 0.001), with increases in latency over age (Figure 2). The p13 latency for the 3 and 4–6 month groups was significantly shorter than for ages 7+ months (all *p* < 0.05). Latencies of the 7–12 month, 1–3, and 4–6 year groups were shorter than those aged 7+ years (all *p* < 0.05). For individuals younger than 7, p13 latency was shorter than adult levels. The n23 latency of the 3-month group was significantly shorter than older age groups (all *p* < 0.05). Additionally, latencies for the 4–6, 7–12 month, and 1–6 year groups remained shorter than those aged 7+ years (all *p* < 0.05), and age-related increases continued until reaching adult levels around 7–12 years (Table 2).

### 3.4. Amplitude and IAR of ACS-cVEMP and BCV-cVEMP in Different Age Groups with Normal Hearing

**Figure 3:**
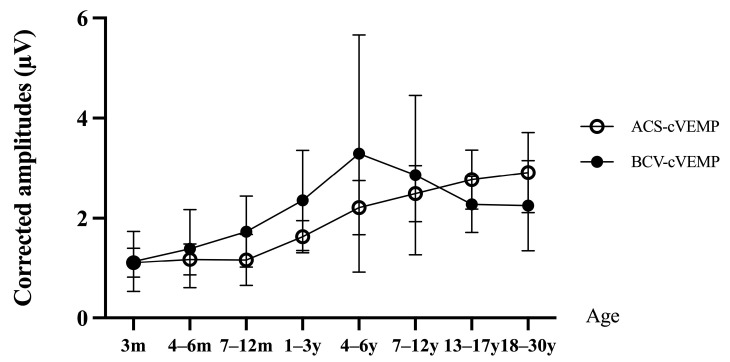
Corrected amplitudes of ACS-cVEMP and BCV-cVEMP among different age groups with normal hearing. Open circles represent corrected amplitudes of ACS-cVEMP. Solid black circles represent corrected amplitudes of BCV-cVEMP. In normal hearing individuals across different age groups, the corrected amplitudes of ACS-cVEMP gradually increase with age, while BCV-cVEMP corrected amplitudes initially increase with age, peak at ages of 4–6 years old, and then gradually decrease with older age.

ACS-cVEMP amplitudes increased with age, with no significant difference among the 3, 4–6, and 7–12 month groups (all *p* > 0.05). However, these groups showed significantly lower amplitudes than those aged 1+ years (all *p* < 0.05). By age 4, amplitudes reached adult levels, indicating smaller amplitudes in infants under 4 (Figure 3). In contrast, BCV-cVEMP amplitudes increased in younger age groups, peaking at 4–6 years before gradually declining. Amplitudes were significantly smaller in the 3-month group compared to those aged 7+ months (all *p* < 0.05). For ages 7–12 months, amplitudes were lower than those aged 1+ years but did not differ significantly from adult levels. The normal reference ranges for p13 and n23 latencies and amplitudes of ACS-cVEMP and BCV-cVEMP in different age groups with normal hearing are shown in Table 3.

**Figure 4:**
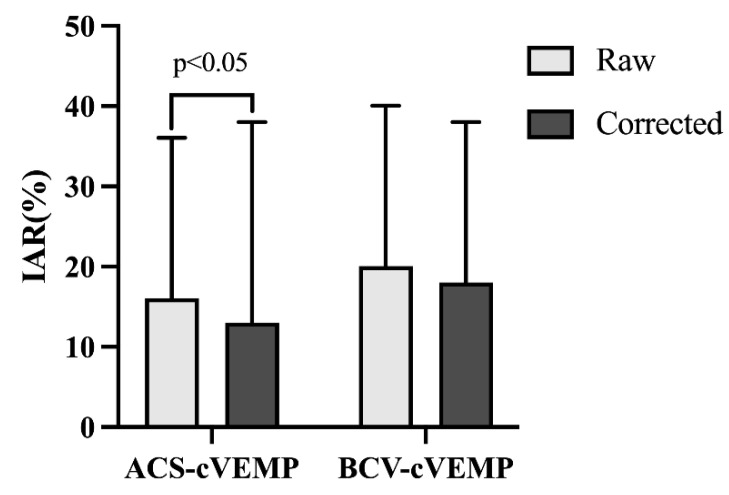
Raw and corrected IAR of ACS-cVEMP and BCV-cVEMP in all groups with normal hearing. Left Side Bar Graph Legend: Light gray bars represent uncorrected ACS-cVEMP amplitudes. Dark gray bars represent corrected ACS-cVEMP amplitudes. Right Side Bar Graph Legend: Light gray bars represent uncorrected BCV-cVEMP amplitudes. Dark gray bars represent corrected BCV-cVEMP amplitudes. Corrected IAR values for ACS-cVEMP are lower than raw IAR values, while there is no statistically significant difference between corrected and raw IAR values for BCV-cVEMP.

There were significant differences in the raw and corrected IARs of ACS-cVEMP. Corrected IARs were significantly lower than raw IARs (*p* = 0.001), while BCV-cVEMP IARs remained stable before and after correction (*p* = 0.452), as shown in Figure 4.

## 4. Discussion

This study validates that ACS-cVEMP and BCV-cVEMP yield comparable response rates across different age groups, supporting cVEMP as a valuable early assessment tool for vestibular function. Key cVEMP parameters, including response rate, latency, interpeak interval, and amplitude, are essential indicators of sacculocollic reflex maturation [12]. Understanding vestibular development in early childhood is critical for identifying potential abnormalities.

Notably, this study is the first to systematically stratify 96 normal-hearing infants aged 3 months to 3 years into detailed age groups (31 in the 3-month group, 25 in the 4–6 months group, 20 in the 7–12 months group, and 20 in the 1–3 years group). This detailed age stratification and well-defined sample size exceed those of previous studies, providing a more precise and reliable insight into vestibular development during this crucial period.

### 4.1. The Response Rates in Different Age Groups with Normal Hearing

Our study found higher cVEMP response rates in normal hearing infants (ACS-cVEMP: 87–100%; BCV-cVEMP: 97–100%) compared to other studies. Verrecchia et al. [14] reported a 78.4% BCV-cVEMP response rate in 1–6 month-olds, possibly due to several factors: (1) Positioning: In Verrecchia et al. [14], infants were supine and held by parents, with the examiner lifting the head and assisting with neck rotation. In our study, infants were placed supine on the bed, and the examiner assisted with lifting and rotating the head while parents held the shoulders to prevent movement. This position might result in higher electromyography (EMG) level, facilitating waveform response. (2) Bone Vibrators: The B-81 bone vibrator used here has greater output power and less distortion compared to the commonly used B-71 [14,24,25], enhancing low-frequency response and better suitability for VEMP response [26]. (3) Operator Skill: experienced and skilled examiners can more accurately control test conditions, such as the placement of headphones/bone vibrators and the subject’s position, thereby improving response rates.

We also observed that BCV-cVEMP response rates were higher than that of ACS-cVEMP in younger infants. Several factors might contribute to this: (1) Different conduction pathways: ACS transmit through the external and middle ear, which can be affected by vernix and cerumen in young infants, impacting sound delivery. BCV directly stimulates vestibular organs, bypassing the external and middle ear. (2) Headphone-induced external auditory canal collapse: In young infants, headphones might cause the external auditory canal to collapse, affecting sound transmission [27,28]. (3) Different stimulation mechanisms: BCV causes linear acceleration of the skull, whereas ACS induces fluid movement in the labyrinth through stapes vibration, thus potentially activating more nerve fibers with BCV [29,30,31]. Another hypothesis is that otolithic hair cells respond differently to ACS and BCV stimulation. BCV causes relative motion between the cell body and cilia, while ACS causes relative motion of the cilia to the cell body, with BCV generating more effective shearing motion on the otolithic membrane, leading to the activation of more hair cells [31]. However, the exact mechanism requires further research.

### 4.2. Latencies in Different Age Groups with Normal Hearing

Our study observed age-related increases in ACS-cVEMP and BCV-cVEMP latencies, with ACS reaching adult levels at 13–17 years and BCV at 7–12 years, consistent with other findings [22,32,33]. Maes et al. [34] reported that children aged 4–13 years old had shorter p13 and n23 latencies in ACS-cVEMP than adults. Kelsch et al. [18] found that children aged 3–5 years had significantly shorter ACS-cVEMP latencies than children older than 5 years old, with no significant difference in latencies among the 6–7, 8–9, and 10–11 years old groups, suggesting that shorter neck length in younger children may result in shorter reflex pathways. However, their study did not include an adult group, so the difference between children and adults could not be determined. Some studies suggest that neck length can be used as a proxy for estimating reflex pathway length, with a positive correlation between neck length and cVEMP latency when the neck length is less than 15.3 cm beyond which the results are similar to adults [35,36]. It has been reported that factors such as the degree of myelination, synaptic transmission efficiency, reflex pathway length, nerve fiber inclination, and conduction velocity can all influence latency [21,37,38,39]. As age increases, the reflex pathway gradually matures, and the increase in conduction velocity compensates for the increased conduction path length, resulting in latencies approaching adult levels [2,8,17,20,40]. The variability in reported data across studies may be attributed to several factors. Firstly, different age ranges and group spans in studies contribute to inconsistencies. For instance, Marten’s study grouped infants aged 5–12 months together [24], whereas our study had more detailed age stratification. Secondly, differences in testing protocols, recording techniques, and parameter settings also impact results, highlighting the importance of establishing standardized reference values for each age group.

### 4.3. Amplitudes and IAR in Different Age Groups with Normal Hearing

Numerous studies have established that cVEMP amplitude has a linear relationship with EMG level [22,41]. Controlling the bilateral EMG level can further reduce intra- and inter-individual amplitude differences, enhancing diagnostic sensitivity [42]. To avoid amplitude differences caused by EMG asymmetry, we calibrated the EMG level of each subject and standardized the amplitude. Our results showed no significant difference in ACS-cVEMP amplitudes among groups over 4 years of age, while amplitudes increased with age before this period. This could be related to the testing methods and the level of cooperation. Studies [43] indicated that minor changes in headphone placement can lead to waveform disappearance or reduction. Unlike adults, infants cannot actively turn their heads to contract the SCMM. During testing, an audiologist lifted and turned the infants’ heads, during which the infants might resist and cry, causing the headphones and bone vibrator to shift, reducing amplitude, while children over 4 years old can actively cooperate with the test. Valente et al. [16] compared parameter results between children aged over 3 years old and adults, finding no significant difference in corrected amplitudes, indicating that the VCR pathway is fully developed by 3 years, consistent with our study. Studies also reported that EMG increases with age within a certain range [44]. Although amplitude correction can mitigate the impact of EMG to some extent, this correction is only effective within a certain range. When EMG levels are too low, waveforms may be difficult to elicit, or amplitudes may be reduced. Conversely, excessively high EMG levels can make background noise difficult to separate, affecting signal averaging and thereby reducing corrected amplitudes. Excessively high EMG levels may also lead to amplitude saturation, where corrected amplitudes no longer significantly change with increasing EMG levels [44].

Unlike ACS-cVEMP, BCV-cVEMP amplitudes initially increase with age, peaking at 4–6 years old, and then gradually decrease with further aging. Vacher et al. [22] similarly found that BCV-cVEMP amplitudes increase from birth to 4–6 years, then slowly decrease. This phenomenon may be related to anatomical changes in the skull during infant growth. Compared with adults, infants have smaller external auditory canal volumes, resulting in higher sound pressure levels for the same ACS stimulation intensity [45,46]. For bone conduction, smaller external auditory canals may cause an occlusion effect at low frequencies, increasing BCV stimulation intensity [46]. As age increases, the thickness of the cortical bone may reduce BCV conduction efficiency [27]. These factors collectively contribute to the age-related changes in amplitude. Additionally, differences in testing positions, age ranges, equipment, and parameter settings can impact results, emphasizing the need for each center to establish normal reference ranges for specific age groups. Using data from other centers without adjustment could lead to inaccurate result interpretations.

Our results showed that the corrected IAR values for ACS-cVEMP were significantly lower than the raw IARs, while BCV-cVEMP IARs were unaffected by EMG correction. These differences may be due to EMG level control for BCV-cVEMP (50–200 μV) while ACS-cVEMP only used the instrument’s built-in amplitude correction without EMG level control. Previous studies suggest IAR decreases with controlled EMG or amplitude correction, though not always statistically significantly [47], which could be related to the fact that subjects in that study were all normal young adults who presented with ideal EMG levels and amplitudes. It was reported that when SCMM contraction meets testing requirements, the significance of correction diminishes. Additionally, some subjects’ IAR values changed from normal to abnormal after EMG correction, which required further investigation. Therefore, caution is advised when interpreting IARs in infants, as factors like testing conditions and cooperation level may affect accuracy. Follow-up evaluations are recommended to confirm unilateral dysfunction.

## 5. Conclusions

In summary, our study demonstrates that cVEMP testing, including both ACS-cVEMP and BCV-cVEMP, is feasible even in infants as young as 3 months, supporting its potential role in early vestibular screening. The establishment of age-specific normative data enhances diagnostic accuracy by allowing clinicians to differentiate between age-appropriate responses and potential abnormalities. Early identification of vestibular dysfunction may be crucial in preventing delays in motor development, balance control, and spatial orientation. Therefore, the incorporation of cVEMP screening in routine audiological or pediatric assessments, particularly in populations at risk (e.g., children with congenital hearing loss or delayed milestones), could facilitate timely interventions and improve developmental outcomes.

However, this study has several limitations. Specifically, our study employed a cross-sectional design, which limits the ability to track individual developmental trajectories over time. Additionally, while we included a relatively wide age range, the sample size within certain younger age subgroups was limited, which may affect the generalizability of the normative data. Future longitudinal studies with larger, more diverse cohorts are warranted to validate our findings and to explore the influence of factors such as sex, motor development, and environmental influences on vestibular maturation. Furthermore, incorporating other vestibular function tests at appropriate ages could offer a more comprehensive understanding of vestibular system development.

Overall, this study lays a foundation for the clinical application of vestibular screening in early childhood and highlights the importance of early identification and management of vestibular deficits in pediatric populations.

## Figures and Tables

**Table 1:** The distribution of age, gender, and number of subjects in different age groups.

Age Group	Number of Subjects	Average Age (Mean ± SD)	Gender (Male/Female)
3 months	31	3.0 ± 0.00	19/12
4–6 months	25	5.12 ± 0.85	12/13
7–12 months	20	9.05 ± 1.74	10/10
1–3 years	20	24.20 ± 8.96	12/8
4–6 years	20	58.80 ± 9.33	10/10
7–12 years	23	105.91 ± 19.15	13/10
13–17 years	20	180.00 ± 13.86	10/10
18–30 years	20	250.80 ± 27.15	10/10

**Table 2:** ACS-cVEMP and BCV-cVEMP parameters in different age groups with normal hearing.

	Age Group	Number of Ears	p13 Latency (ms)	n23 Latency (ms)	p13–n23 Interval (ms)	Corrected Amplitude (μV)
ACS-cVEMP	3 months	54	12.48 ± 0.92	18.08 ± 0.84	5.60 ± 0.95	1.11 ± 0.29
	4–6 months	44	12.61 ± 0.97	18.47 ± 1.09	5.86 ± 1.04	1.18 ± 0.31
	7–12 months	38	12.64 ± 0.72	19.59 ± 0.73	6.95 ± 0.98	1.17 ± 0.51
	1–3 years	40	13.39 ± 0.85	20.05 ± 0.90	6.66 ± 1.01	1.63 ± 0.32
	4–6 years	40	13.55 ± 1.27	20.71 ± 1.55	7.16 ± 1.67	2.21 ± 0.54
	7–12 years	46	13.42 ± 0.92	21.39 ± 0.86	7.96 ± 1.01	2.49 ± 0.56
	13–17 years	40	14.43 ± 1.12	22.05 ± 0.82	7.62 ± 1.10	2.77 ± 0.59
	18–30 years	40	14.47 ± 1.08	22.29 ± 0.92	7.82 ± 1.14	2.91 ± 0.80
	*p* value		<0.001	<0.001	<0.001	<0.001
	F value		26.072	140.497	34.606	97.983
BCV-cVEMP	3 months	60	11.95 ± 0.92	21.09 ± 1.44	9.14 ± 1.10	1.14 ± 0.60
	4–6 months	48	12.16 ± 0.85	21.89 ± 1.81	9.74 ± 1.58	1.39 ± 0.78
	7–12 months	40	13.26 ± 1.76	22.28 ± 1.56	9.02 ± 1.63	1.73 ± 0.71
	1–3 years	40	13.72 ± 1.19	22.28 ± 1.43	8.56 ± 0.97	2.36 ± 1.00
	4–6 years	40	13.35 ± 0.91	22.23 ± 1.01	8.88 ± 1.01	3.29 ± 2.37
	7–12 years	46	15.71 ± 1.48	24.11 ± 1.18	8.39 ± 1.28	2.86 ± 1.59
	13–17 years	40	15.25 ± 1.13	24.31 ± 1.29	9.06 ± 1.39	2.28 ± 0.56
	18–30 years	40	15.57 ± 1.32	24.61 ± 1.32	9.04 ± 1.28	2.25 ± 0.90
	*p* value		<0.001	<0.001	<0.001	<0.001
	F value		80.310	42.342	4.063	23.998

**Table 3:** Normal reference ranges for ACS-cVEMP and BCV-cVEMP in different age groups with normal hearing.

Age Group	ACS-cVEMP			BCV-cVEMP		
	p13 Latency (ms)	n23 Latency (ms)	Corrected Amplitude (μV)	p13 Latency (ms)	n23 Latency (ms)	Corrected Amplitude (μV)
3 months	12.24–12.73	17.85–18.30	1.03–1.19	11.72–12.19	20.72–21.46	0.99–1.30
4–6 months	12.33–12.89	18.16–18.79	1.09–1.27	11.91–12.40	21.37–22.42	1.16–1.61
7–12 months	12.40–12.88	19.35–19.83	1.01–1.34	12.70–13.82	21.79–22.78	1.50–1.95
1–3 years	13.12–13.66	19.76–20.33	1.52–1.73	13.34–14.10	21.83–22.74	2.04–2.68
4–6 years	13.14–13.96	20.21–21.20	1.85–2.15	13.06–13.65	21.91–22.56	2.53–4.04
7–12 years	13.15–13.70	21.13–21.64	2.32–2.65	15.27–16.15	23.76–24.46	2.39–3.33
13–17 years	14.07–14.79	21.79–22.31	2.58–2.96	14.89–15.62	23.90–24.72	2.10–2.46
18–30 years	14.13–14.82	22.00–22.58	2.66–3.17	15.15–16.00	24.19–25.03	1.96–2.54

## Data Availability

All data used during the study are available from the corresponding author by request.
